# Determination of the Metabolite Content of *Brassica juncea* Cultivars Using Comprehensive Two-Dimensional Liquid Chromatography Coupled with a Photodiode Array and Mass Spectrometry Detection

**DOI:** 10.3390/molecules25051235

**Published:** 2020-03-09

**Authors:** Katia Arena, Francesco Cacciola, Laura Dugo, Paola Dugo, Luigi Mondello

**Affiliations:** 1 Department of Chemical, Biological, Pharmaceutical and Environmental Sciences, University of Messina, 98122 Messina, Italy; arenak@unime.it (K.A.); pdugo@unime.it (P.D.); lmondello@unime.it (L.M.); 2 Department of Biomedical, Dental, Morphological and Functional Imaging Sciences, University of Messina, 98122 Messina, Italy; 3 Department of Sciences and Technologies for Human and Environment, University Campus Bio-Medico of Rome, 00128 Rome, Italy; 4 Chromaleont s.r.l., c/o Department of Chemical, Biological, Pharmaceutical and Environmental Sciences, University of Messina, 98122 Messina, Italy; 5 BeSep s.r.l., c/o Department of Chemical, Biological, Pharmaceutical and Environmental Sciences, University of Messina, 98122 Messina, Italy

**Keywords:** *Brassica juncea* spp., matabolites, foods, comprehensive two-dimensional liquid chromatography, mass spectrometry, multi segmented-in-fraction gradient

## Abstract

Plant-based foods are characterized by significant amounts of bioactive molecules with desirable health benefits beyond basic nutrition. The Brassicaceae (Cruciferae) family consists of 350 genera; among them, *Brassica* is the most important one, which includes some crops and species of great worldwide economic importance. In this work, the metabolite content of three different cultivars of *Brassica juncea*, namely ISCI Top, “Broad-leaf,” and ISCI 99, was determined using comprehensive two-dimensional liquid chromatography coupled with a photodiode array and mass spectrometry detection. The analyses were carried out under reversed-phase conditions in both dimensions, using a combination of a 250-mm microbore cyano column and a 50-mm RP-Amide column in the first and second dimension (^2^D), respectively. A multi (three-step) segmented-in-fraction gradient for the ^2^D separation was advantageously investigated here for the first time, leading to the identification of 37 metabolites. In terms of resolving power, orthogonality values ranged from 62% to 69%, whereas the corrected peak capacity values were the highest for *B. juncea* ISCI Top (639), followed by *B. juncea* “Broad-leaf” (502). Regarding quantification, *B. juncea* cv. “Broad-leaf” presented the highest flavonoid content (1962.61 mg/kg) followed by *B. juncea* cv. ISCI Top (1002.03 mg/kg) and *B. juncea* cv. ISCI 99 (211.37 mg/kg).

## 1. Introduction

Vegetables from the Brassicaceae or Cruciferae family represent the most commonly consumed vegetables worldwide. This family includes brussels sprouts, broccoli, cabbage, cauliflower, and others. Such vegetables do contain high levels of bioactive compounds, e.g., polyphenols, carotenoids, tocopherols, glucosinolates, and ascorbic acid [1,2,3,4]. Epidemiological data have demonstrated the ability of *Brassica* vegetables to decrease the risk of cardiovascular diseases and several types of cancer, e.g., such as in the gastrointestinal tract [5]. All these effects have been associated with the presence of bioactive molecules with antioxidant and free radical scavenging properties, with potential effects on gene expression, cell signaling, and cell adhesion [6].

Among the bioactive compounds that occur in the Brassicaceae family, polyphenols represent a group of secondary plant metabolites comprising diverse families [7]. Among them, the most common subclass of polyphenols is represented by flavanols, and the most abundant aglycones are quercetin and kaempferol, which often occur as a complex conjugated via glycosilation and acylation of the aglycone [7]. Frequently, these compounds occur in acylated forms with hydroxycinnamic acids; among them, the most abundant are *p*-coumaric acid, caffeic acid, ferulic acid, and sinapic acids. These complex compositions are influenced by many factors, e.g., cultivar, climate, postharvest treatments, and agricultural and environmental variables [8,9,10,11].

Belonging to the Brassicaceae family, *Brassica juncea* L. is an amphiploid species, mainly grown as a food crop but is also used for medicinal purposes. It is one of the richest sources of iron, vitamin A, and vitamin C but also contains potassium, calcium, thiamine, riboflavin, and 𝛽-carotene. It has antiseptic, diuretic, emetic, and rubefacient properties. It has been reported to contain antioxidants like flavonoids, carotenes, lutein, indoles, and zeaxanthin [12].

The metabolite profiling of *Brassica juncea* L. has up until now been carried out using high- or ultra-high-performance liquid chromatography (HPLC, UHPLC) coupled with photodiode array (PDA) and/or MS detection [13,14,15,16,17,18]. However, due to its complexity, related to the simultaneous presence of isobaric molecules, a single separation system *viz.* one-dimensional LC (1D–LC) hampers a full profiling of such complex samples, thus negatively affecting quantification data [14,15,16]. An alternative expedient for overcoming such an issue could be the use of advanced analytical tools, e.g., comprehensive two-dimensional LC (LC×LC). The latter, which comprises two orthogonal separation mechanisms, can provide higher resolving power, which is the peak capacity (n_C_) multiplicative of the peak capacity values in both dimensions [19,20,21,22,23,24,25,26,27,28,29,30,31,32].

The likelihood of achieving “orthogonal” separation mechanisms in LC×LC separations is quite high considering the hydrophobicity, polarity, size and charge; however, some technical difficulties can arise as a result of the chosen coupling. As an example, a combination of normal phase (NP) and reversed phase (RP) may lead to the precipitation of buffers or salts due to the mobile phase immiscibility [26,27]. On the other hand, when dealing with RP-LC×RP–LC separations, no solvent compatibility issues are usually observed. The main issue for such a set-up, using a conventional “full in fraction” in the second dimension (^2^D) gradient, is the limited orthogonality due the similarity of the stationary phases employed. In fact, the analytes tend to align themselves along the diagonal line in the 2D contour plot. To ameliorate such an issue, some expedients (tailored gradient programs) have been exploited in recent years [19,20,21,25,30,31,32]. A first type of gradient is called a “segmented gradient,” where at least two different gradient segments are employed throughout the whole RP–LC×RP–LC run. As a result, a remarkable bandwidth suppression effect is achieved and the likelihood of the “wrap-around” phenomena is toned down [19,21,22]. A second type of gradient, a parallel gradient mode, involves a single ^2^D gradient run matching the ^1^D one. In this case, a longer ^2^D elution time can be used since a post-gradient equilibration is not necessary [19,21]. Also, the use of “shift gradients” for the ^2^D run has been proposed with the aim to adopt a (changing) narrower gradient program for the entire RP-LC×RP-LC analysis time. This approach turned out to be a very effective one in various natural product and food applications [20,25,30,31,32].

In this contribution, a newly developed RP-LC×RP–LC system coupled with PDA and MS detection for the untargeted metabolite content of three different cultivars of *Brassica juncea*, namely ISCI 99, ISCI Top and “Broad-leaf,” is reported. Separations were conducted using a combination of a first dimension (^1^D) microbore cyano column, and a ^2^D superficially porous RP-Amide column. A novel ^2^D gradient mode, namely a multi (three-step) segmented-in-fraction gradient, was proposed and successfully demonstrated, leading to an improved expansion of metabolic coverage.

## 2. Results and Discussion

The analysis of the three different cultivars of *B. juncea* L. was first run using a conventional LC-PDA-MS approach on a C18 column. As illustrated in the following section, a considerable number of compounds overlapped; consequently, an RP–LC×RP–LC system was adopted in order to attain higher separation power, thus providing a thorough overview of the overall metabolites pool, which is beneficial for quantification purposes.

### 2.1. Elucidation of Brassica juncea Cultivars Using RP-LC×RP-LC-PDA-MS

**Figure 1:**
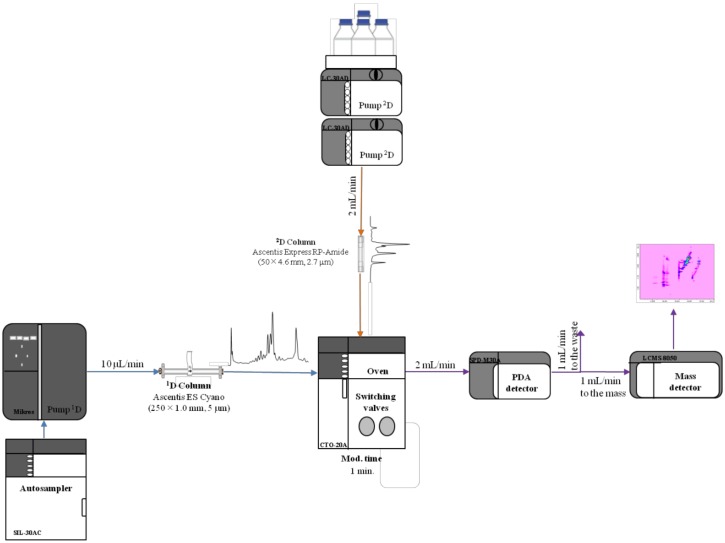
Scheme of the reversed-phase liquid chromatography RP-LC×RP-LC system employed for the investigated work. PDA: Photodiode array.

RP-LC×RP-LC separations have proved to be quite effective for the analysis of the metabolite content of food and natural products [19,20,21,22,25,30,31,32]. Before running an RP-LC×RP-LC analysis, a careful optimization of the independent separations must be carried out [26,27,29]. A low mobile phase flow rate is preferred in the ^1^D in order to decrease the fraction volume onto the ^2^D and augment the ^1^D sampling rate. Usually, this is achieved by employing a microcolumn in the ^1^D; however, since most commercial LC pumps are not capable of delivering a stable and repeatable flow rate, a higher flow rate is commonly employed and split up before entering the ^1^D column. A scheme of the RP-LC×RP-LC employed is reported in Figure 1.

In this work, a robust and easy-to-use micropump with a completely new direct-drive engineering was advantageously employed, and was capable of delivering micro- to semi-micro flow rates ranging from 1 to 500 μL/min. Repeatability data obtained on four selected peaks are displayed in Table 1. Relative standard deviation (RSD, %) values lower than 0.02 were attained in the case of mean retention times (min), whereas RSD (%) values lower than 1.21 were determined in case of mean areas.

With regard to the ^2^D, a fast separation is commonly employed in order to increase the ^1^D sampling to lower the risk of incurring wrap-around phenomena. Consequently, a microcyano column was chosen in the ^1^D, whereas a 4.6-mm I.D. partially porous RP-Amide column was employed in the ^2^D and operated at 2 mL min^−1^. For fraction transfer, two high-speed, six-port, two-position switching valves equipped with two 10 µL sampling loops were chosen.

In this context, the optimization of the gradient programs, especially for the ^2^D, is also necessary for an adequate separation and is mainly related to the chemical properties of the solutes. Late eluting compounds that are retained more in the ^2^D require a greater gradient steepness in order not to incur wrap-around effects. In the case of closely related compounds, e.g., early-eluting compounds, which are subjected to co-elutions, a lower gradient of steepness is preferable in order to permit stronger retention.

**Figure 2:**
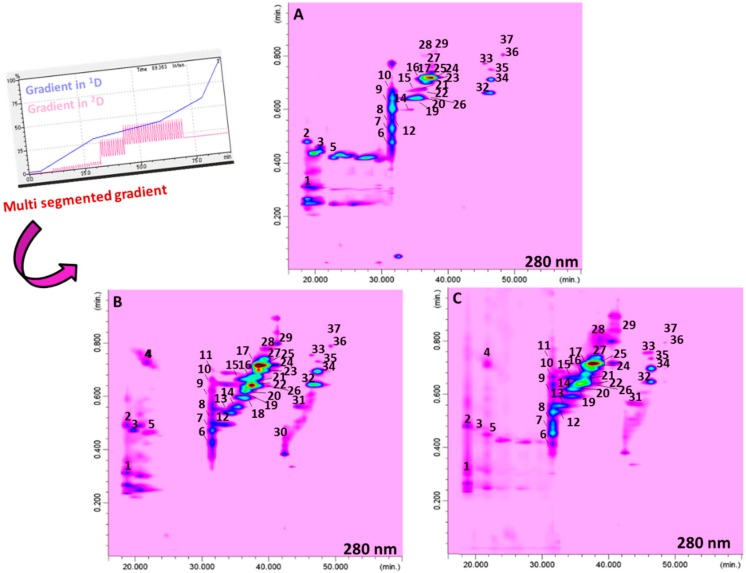
RP-LC×RP-LC contour plots of the system employed for ISCI Top (**A**), “Broad-leaf“ (**B**), and ISCI 99 (**C**).

Following this strategy, a newly developed RP-LC×RP-LC system was investigated. In particular, a multi segmented-in-fraction gradient approach was employed, as illustrated in Figure 2. In particular, three different full-in-fraction gradients were considered for the ^2^D analysis. The first gradient was from 10 to 32 min, where %B ranged from 3% to 8% (Δ%B: 5) for the analysis of early eluting organic acids; in the second gradient step (from 32 to 43 min), %B ranged from 10% to 44% (Δ%B: 34) for the analysis of (acetylated) tri- and tetrasaccharides, whereas in the last one (from 43 to 60 min), %B ranged from 20% to 60% (Δ%B: 40) for the analysis of late eluting (acetylated) mono- and disaccharides. The modulation time of the switching valves was 1.00 min.

Figure 2 shows the contour plots for the RP-LC×RP-LC analysis of the three cultivars of *Brassica juncea,* where a total of 37, 34, and 31 metabolites were positively separated using the optimized multi segmented-in-fraction gradient approach.

Concerning the performance of the developed RP-LC×RP-LC system, Table 2 reports the values attained for both peak capacity and orthogonality [33]. The highest theoretical peak capacity values, which are multiplicative of the peak capacity of the two single dimensions [34], were attained for the cultivar ISCI Top (1734), whereas the lowest was obtained for the cultivar ISCI 99 (932). The orthogonality values ranged from 62% to 69% for ISCI Top and “Broad leaf”, respectively [33]. The corrected peak capacity values, which considered, both undersampling [35] and orthogonality values, were 639, 404, and 502 for ISCI Top, ISCI 99, and “Broad leaf”, respectively. Considering the similarity of the two separation systems employed in both dimensions, such values can be considered quite remarkable and are in agreement with previously published findings on similar set-ups exploited for polyphenolic characterization in licorice (695 in Wong et al. [30]) and pistachio (461–633 in Arena et al. [31]) samples.

**Figure 3:**
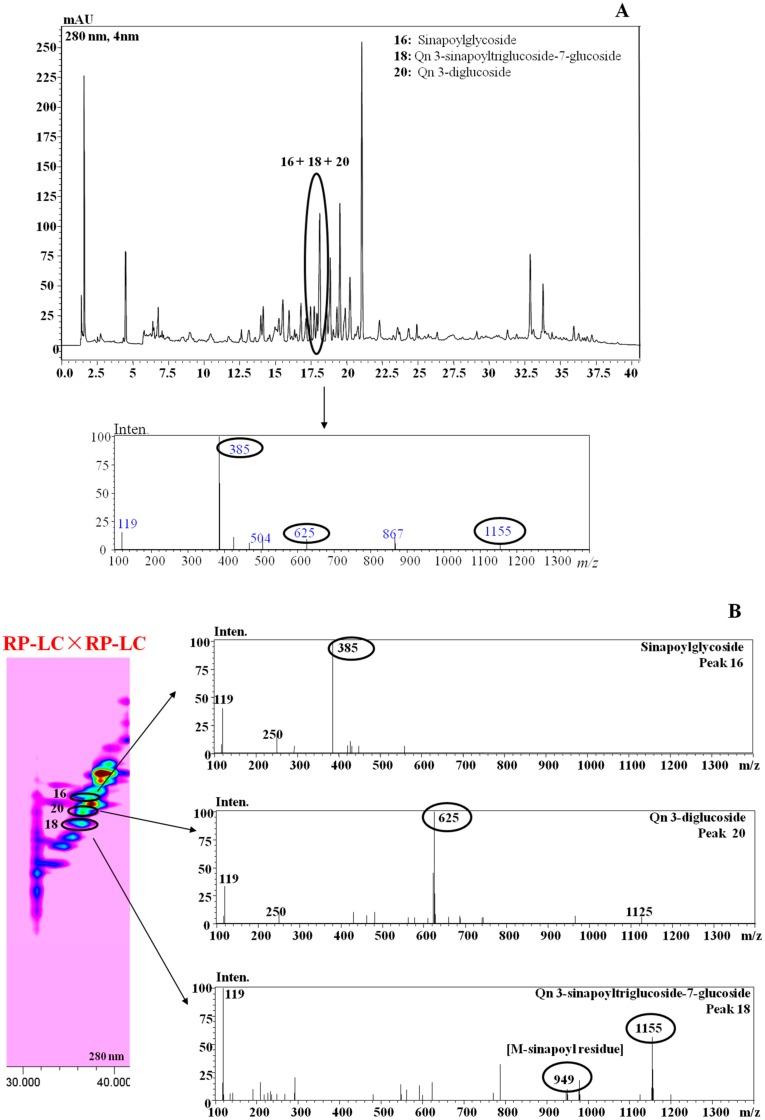
RP-LC (**A**) vs. RP-LC×RP-LC (**B**) analysis of metabolites in *B. juncea* cv. ISCI Top. Qn: Quercetin.

As an example, the benefits associated with the employment of the developed RP-LC×RP-LC with the multi segmented-in-fraction gradient program over the conventional RP-LC separation are highlighted in Figure 3.

A selected chromatographic region of the *Brassica* ISCI Top extract (Figure 3A) clearly shows how the 1D-LC did not provide enough peak capacity for unambiguous characterization of the chemical profile of the three occurring metabolites, due to compound overlapping. However, when the RP-LC×RP-LC analysis was employed, the three different bioactive compounds were conveniently separated and characterized via inspection of the respective MS spectra (Figure 3B). As a result, the better resolution of the RP-LC×RP-LC separation (with the ^2^D operated under the multi (three-step) segmented-in-fraction gradient mode) over the conventional 1D-LC led to a greater metabolite expansion in the RP-LC×RP-LC space, which was essential for improving the reliable identification of compounds with complexity and/or various polarities.

### 2.2. Semi-Quantitative Determination of the Flavonoid Content of Brassica juncea Cultivars

Tentative identification of the *Brassica juncea* extracts, illustrated in Figure 2, was performed based on their PDA, MS, and literature data [1,2,9,10,11,14,15,16,36,37,38]. Among the major classes of compounds identified, organic acids, (acetylated) tri- and tetrasaccharides, and (acetylated) mono- and disaccharides, were recognized (Table 3). Due to the lack of commercial standards, quantification of *Brassica* spp content has so far been carried out after acidic and/or alkaline hydrolysis [36,37,38]. In this work, a quantification of the native flavonoid composition of the three cultivars of *Brassica juncea* was carried out by RP-LC×RP-LC system coupled to PDA detection for the first time. Toward such an aim, and considering the unavailability of corresponding standard references, an established approach in the field of food and natural product analysis was followed. Basically, three standards, as representatives of the distinct chemical classes, i.e., Km 3-*O*-glucoside, Isorhamnetin (Is) 3-*O*-glucoside, and Qn 3-*O*-glucopyranoside, were chosen and calibration curves were prepared, as reported in Section 3.4.5. Results are shown in Table 4, which reports all the standard curves, correlation coefficients (R^2^), limits of detection (LoDs) and limits of quantification (LoQs), and relative standard deviations (RSDs) of the peak areas for each standard selected. The five-point calibration curves provided R^2^ values ranging from 0.9993 to 0.9997, whereas for LoQ and LoD, values as low as only 30 ppb and 90 ppb, respectively, were found. Finally, RSD values lower than 0.89% were obtained, demonstrating valuable method repeatability.

**Figure 4:**
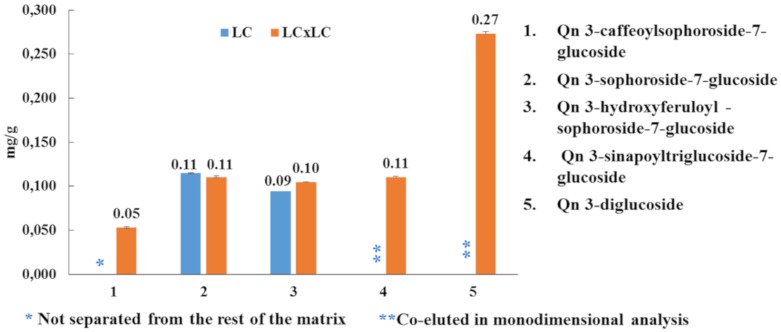
Semi-quantitative results (mg/g) for quercetin derivates using RP-LC and RP-LC×RP-LC-PDA.

Subsequently, all three samples were analyzed and the contents of the target compounds were calculated using commercially-available software, as reported in Table 3. *B. juncea* cv. “Broad-leaf” presented the highest flavonoid content (1962.61 mg/kg), followed by *B. juncea* cv. ISCI Top (1002.03 mg/kg) and *B. juncea* cv. ISCI 99 (211.37 mg/kg). Is 3,7-diglucoside turned out to be the most abundant flavonoid in each cultivar investigated (ISCI Top: 309.48 mg/kg; “Broad-leaf”: 1321.50 mg/kg; ISCI 99: 130.2 mg/kg), followed by Km 3-sinapoylsophorotrioside-7-glucoside (ISCI Top: 284.54 mg/kg; “Broad leaf”: 174.38 mg/kg; ISCI 99: 10.88 mg/kg). Considering the three different flavonoid classes, isorhamnetin derivates were the most abundant flavonoids in the cultivars “Broad-leaf” and ISCI 99 (1366.91 mg/kg vs. 136.83 mg/kg); on the other hand, with regard to the cultivar ISCI Top, kaempferol derivates were detected in the highest amount (598.65 mg/kg). Interestingly, as an example, looking at the quercetin derivates, by using the LC×LC technique, it was possible to quantify all of them, unlike the conventional LC, in which some of the compounds could not be determined due to either co-elutions or matrix interferences (Figure 4).

## 3. Materials and Methods

### 3.1. Chemical and Reagents

LC-MS-grade water, methanol, acetonitrile, and acetic acid were obtained from Merck Life Science (Merck KGaA, Darmstadt, Germany). Km 3-*O*-glucoside, Is 3-*O*-glucoside, and Qn 3-*O*-glucoside were obtained from Merck Life Science (Merck KGaA, Darmstadt, Germany). Stock solutions of 1000 mg L^−1^ were prepared for each standard by dissolving 10 mg in 10 mL of methanol.

1D-LC separations were performed on an Ascentis Express C18 column (Merck Life Science, Merck KGaA, Darmstadt, Germany; 150 × 4.6 mm I.D., 2.7 μm dp). LC×LC separations were conducted by using a ^1^D Ascentis ES-Cyano (ES-CN) column (Merck Life Science, Merck KGaA, Darmstadt, Germany; 250 × 1.0 mm I.D., 5 μm dp) and a ^2^D Ascentis Express RP-Amide column (Merck Life Science, Merck KGaA, Darmstadt, Germany; 50 × 4.6 mm I.D., 2.7 μm dp).

### 3.2. Sample and Sample Preparation

*Brassica juncea* L. Czern & Coss cv. ISCI 99, ISCI Top, and “Broad-leaf” leaf selections were provided from the *Brassica* collection of Consiglio per la ricerca in agricoltura e l’analisi dell’economia agraria – Centro di Ricerca Cerealicoltura e Colture Industriali) (CREA-CI) [39]. Samples were immediately frozen and freeze-dried for storage in glass vacuum desiccators. Lyophilized tissues were finely powdered to 0.5 μm size for analysis. Compound extraction was carried out based on the following protocol [16] with some modifications. The powder of the leaves of the three different *B. juncea* cultivars were weighed into 100 mg samples. The samples were extracted twice with 5 mL of a mixture of methanol:water (60:40, *v/v*) for 30 min in a sonicator and centrifuged at 1000× *g* for 15 min, followed by filtration of the supernatants through a 0.45-µm nylon filter (Merck Life Science, Merck KGaA, Darmstadt, Germany). The prepared organic extracts were subjected to evaporation in a EZ-2 evaporator and then redissolved in 1 mL of the same solvent extraction mixture of methanol:water (60:40, *v/v*).

### 3.3. Instrumentation

LC and LC × LC analyses were performed on a Nexera-e liquid chromatograph (Shimadzu, Kyoto, Japan), consisting of a CBM-20A controller, one LC-Mikros binary pump, two LC-30AD dual-plunger parallel-flow pumps, a DGU-20A_5_R degasser, a CTO-20AC column oven, a SIL-30AC autosampler, and an SPD-M30A PDA detector (1.0 μL detector flow cell volume). The two dimensions were connected by means of two high-speed/high-pressure, two-position, six-port switching valves with a micro-electric actuator (model FCV-32 AH, 1.034 bar; Shimadzu, Kyoto, Japan), placed inside the column oven, and equipped with two 10-µL stainless steel loops. The Nexera-e liquid chromatograph was hyphenated to an LCMS-8050 triple quad mass spectrometer through an ESI source (Shimadzu, Kyoto, Japan).

### 3.4. Analytical Conditions

#### 3.4.1. LC Separations

1D-LC separations were run on the Ascentis Express C18 column. Mobile phases: (A) 0.15% acetic acid in water (pH 3), (B) 0.15% acetic acid in ACN. Gradient: 0 min, 5% B; 5 min, 5% B; 15 min, 10% B; 30 min, 20% B; 60 min, 50% B; 80 min, 100%. Mobile phase flow rate: 1 mL min^−1^. Column oven: 30 °C. Injection volume: 10 µL.

#### 3.4.2. LC×LC Separations

For ^1^D separations, the Ascentis ES-CN column was used. Mobile phases: (A) 0.15% acetic acid in water (pH 3), (B) 0.15% acetic acid in ACN. Gradient: 0 min, 2% B; 5 min, 2% B; 30 min, 30% B; 60 min, 40% B; 80 min, 100% B. Flow rate: 10 μL min^−1^. Column oven: 30 °C. Injection volume: 1 µL.

For ^2^D separations, an RP-Amide column was used. The mobile phases employed were (A) 0.15% acetic acid in water (pH 3), (B) 0.15% acetic acid in ACN. Multi (three-step) segmented-in-fraction gradient conditions: I) 10 to 32 min (cycle: 0.01–0.80 min, 3–8% B; 0.81–1.0 min, 3% B); II) 32 to 43 min (cycle: 0.01–0.80 min, 10–44% B; 0.81–1.0 min, 10% B); III) 43 to 60 min (cycle: 0.01–0.80 min, 20–60% B; 0.81–1.0 min, 20% B). Flow rate: 2 mL min^−1^, modulation time of the switching valves: 1.00 min, loop internal volume: 10 µL, and column oven: 30 °C.

#### 3.4.3. Detection Conditions

PDA range: 200–450 nm; sampling rate: 12.5 Hz (1D-LC analyses), 40 Hz (LC × LC analyses); time constant: 0.08 sec (1D-LC analyses), 0.025 sec (LC × LC analyses).

Interface: ESI-MS in negative ionization mode. Mass spectral range in full scan mode: *m/z* 100–1200; event time: 0.5 (1D-LC analyses), 0.2 sec (LC × LC analyses); nebulizing gas (N_2_) flow: 3 L min^−1^; drying gas (N_2_) flow: 15 L min^−1^; heating gas flow (air): 10 L min^−1^ same; heat block temperature: 400 °C; desolvation line (DL) temperature: 250 °C; interface temperature: 300 °C; interface voltage 3.50 kV; detector voltage: 1.80 kV.

#### 3.4.4. Data Handling

The LC × LC-LCMS-8050 system and the switching valves were controlled using the Shimadzu Labsolution software (ver. 5.93) (Kyoto, Japan). LC×LC-Assist software (ver. 2.00) (Shimadzu, Kyoto, Japan) was used for setting up the multi (three-step) segmented-in-fraction gradient analyses. The LC × LC data were visualized and elaborated into two and three dimensions using Chromsquare ver. 2.3 software (Shimadzu, Kyoto, Japan).

#### 3.4.5. Construction of Calibration Curves

For flavonoid determination, due to the lack of commercial standards, Km 3-*O*-glucoside, Is 3-*O*-glucoside, and Qn 3-*O*-glucopyranoside, as representatives of the distinct chemical classes under evaluation, were selected. Standard calibration curves were prepared in the concentration range 0.1–100 mg L^−1^ with five different concentration levels, run in triplicate. The amount of the compound was finally expressed in mg kg^−1^ of extract.

## 4. Conclusions

In this paper, the benefits associated with the use of a multi (three-step) segmented-in-fraction gradient in the RP-LC×RP-LC-PDA-MS analysis of three *Brassica juncea* cultivars are demonstrated. The coupling of a microcyano and an RP-Amide columns, in the first and second dimension, respectively, provided a characteristic metabolite pattern of the extracts, leading to the identification of 37 bioactives of different chemical nature, i.e., organic acids, (acetylated) tri- and tetrasaccharides, and (acetylated) mono- and disaccharides. Interestingly, the employment of a micro LC pump in the first dimension of the RP-LC×RP-LC-PDA-MS systems allowed for high repeatability and stable retention times and areas. The investigated approach can be advantageously employed for RP-LC×RP-LC metabolic analyses of other complex plant derived extracts.

## Figures and Tables

**Table 1:** Repeatability data calculated from four selected peaks of the RP-LC×RP-LC plots shown in Figure 2. RSD: Relative Standard Deviation. Km: Kaempferol.

No.	Analyte	Mean Tt_R_ (min)	RSD (%) n = 3	Mean Area	RSD (%) n = 3
1	Malic acid	19.32	0.02	59664	0.78
2	Km 3-diglucoside-7-glucoside	34.54	0.02	159713	1.21
3	Sinapoyl-feruloyl-triglucoside	42.73	0.01	95465	0.55
4	Disapoylgentiobiose	47.79	0.01	1481389	1.02

**Table 2:** Peak capacity and orthogonality values for the RP-LC×RP-LC analysis of the three *Brassica juncea* extracts.

Parameter	ISCI Top	ISCI 99	“Broad Leaf”
^1^D peak capacity, ^1^n_C_	51	34	46
^2^D peak capacity, ^2^n_C_	34	28	29
Theoretical peak capacity, ^2D^n_C_	1734	952	1334
Effective peak capacity, ^2D^n_C_	926	652	772
Orthogonality, $A_{0}$	69%	62%	65%
Corrected peak capacity, ^2D^n_C,corr_	639	404	502

**Table 3:** Semi-quantitative analysis (mg/kg) of the flavonoid content of *Brassica spp* extracts. Results are expressed as mean ± S.D. of three replicates.

	Compounds	Molecular Ion [M–H]^-^	λ_max_ (nm)	*B. juncea* cv. ISCI Top	*B. juncea* cv. “Broad Leaf”	*B. juncea* cv. ISCI 99
1	Malic acid	133	215; 260	n.q.	n.q.	n.q.
2	Citric acid	191	215; 260	n.q.	n.q.	n.q.
3	Dihydrocaffeic acid 3-*O*-glucoside	357	250; 323	n.q.	n.q.	n.q.
4	Km 3-dicoumaryl-glucoside	739	202; 257	14.6 ± 2.1	13.1 ± 1.5	-
5	1-methoxyspirobrassin	279	198; 259	n.q.	n.q.	n.q.
6	Qn 3-caffeoylsophoroside-7-glucoside	949	250; 336	5.30 ± 0.1	2.91 ± 0.1	3.01 ± 2.2
7	Qn 3-sophoroside-7-glucoside	787	254; 350	11.02 ± 0.2	23.96 ± 1.0	1.44 ± 0.3
8	Sinapoyl-gentiobiose	547	238; 320	n.q.	n.q.	n.q.
9	Rhamnosyl-ellagic acid 1	447	238; 304	n.q.	n.q.	n.q.
10	Rhamnosyl-ellagic acid 2	447	238; 304	n.q.	n.q.	n.q.
11	Feruloylglucose 1	355	243; 328	n.q.	n.q.	-
12	Km 3-*O*-diglucoside-7-*O*-glucoside	771	263; 330	33.56 ± 0.4	27.70 ± 5.0	3.67 ± 1.9
13	Km 3-sophoroside-7-glucoside	771	266; 343	45.77 ± 1.5	80.29 ± 4.2	-
14	Km 3-caffeoyl-triglucoside-7-glucoside	1095	250, 335	15.97 ± 0.4	21.34 ± 1.4	7.39 ± 0.6
15	Qn 3-hydroxyferuloylsophoroside-7-glucoside	979	250; 337	10.47 ± 0.1	26.62 ± 1.1	4.25 ± 0.9
16	Sinapoylglycoside	385	239, 329	n.q.	n.q.	n.q.
17	Feruloylglucose 2	355	243; 329	n.q.	n.q.	n.q.
18	Qn 3-sinapoyltriglucoside-7-glucoside 1	1155	249; 336	11.05 ± 0.1	-	-
19	Km 3-hydroxyferuloylsophoroside-7-glucoside	963	264; 334	21.21 ± 0.5	8.55 ± 0.3	1.52 ± 1.1
20	Qn 3-diglucoside	625	255; 345	35.75 ± 0.3	61.97 ± 1.9	13.59 ± 1.5
21	Km 3-*O*-caffeoyldiglucoside-7-*O*-glucoside 1	933	249; 335	64.78 ± 0.4	80.29 ± 4.2	7.39 ± 0.6
22	Km 3-sinapoylsophorotrioside-7-glucoside	1139	249; 335	248.54 ± 8.1	174.38 ± 1.9	10.88 ± 0.3
23	Km 3-hydroxyferuloylsophoroside-7-glucoside 2	963	250; 333	27.88 ± 0.6	-	10.57 ± 0.3
24	Km 3-sinapoylsophoroside-7-glucoside	977	266; 333	63.36 ± 0.9	32.92 ± 1.8	1.65 ± 0.2
25	Is 3,7-diglucoside	639	264; 336	309.48 ± 0.4	1321.50 ± 6.3	130.2 ± 4.2
26	Km 3-sinapoylsophorotrioside-7-glucoside	1139	241; 335	29.85 ± 1.7	20.13 ± 1.4	1.88 ± 0.3
27	Km 3-feruloylsophoroside-7-glucoside	947	267; 330	28.57 ± 0.1	19.32 ± 0.7	6.89 ± 0.2
28	Km 3-*O*-coumaroyldiglucoside-7-O-glucoside	917	267;318	4.56 ± 0.1	2.22 ± 0.2	0.41 ± 0.2
29	Sinapoylferuloyltriglucose	885	262; 325	n.q.	n.q.	n.q.
30	Sinapic acid	223	270; 326	n.q.	-	-
31	Sinapoyhydroxyferuloyldiglycoside	739	273; 329	n.q.	n.q.	-
32	Disapoylgentiobiose	753	240; 330	n.q.	n.q.	n.q.
33	Is glycoside	477	254; 348	20.31 ± 0.7	45.41 ± 3.9	6.63 ± 1.3
34	Sinapoyl-feruloylgentiobiose	723	240; 330	n.q.	n.q.	n.q.
35	Diferuloyldiglucoside	693	245; 329	n.q.	n.q.	n.q.
36	Trisinapoylgentiobiose	959	243; 326	n.q.	n.q.	n.q.
37	Feruoyl-disapoyl-gentiobiose	929	246; 329	n.q.	n.q.	n.q.

**Table 4:** Quantitative performance of the polyphenolic reference materials used in this study using the RP-LC×RP-LC system coupled to PDA detection.

Reference Material	Standard Curve	R^2^	LoD (μg/mL)	LoQ (μg/mL)	Precision (RSD, %)
Qn 3-*O*-glucopyranoside	y = 13239x − 9234.3	0.9993	0.03	0.09	0.80
Is 3-*O*-glucopyranoside	y = 1990.7x + 188.37	0.9997	0.12	0.39	0.72
Km 3-*O*-glucopyranoside	y = 4625.7x + 4475.7	0.9994	0.03	0.12	0.89
